# Protocol: Leveraging a demographic and health surveillance system for Covid-19 Surveillance in rural KwaZulu-Natal

**DOI:** 10.12688/wellcomeopenres.15949.2

**Published:** 2020-08-25

**Authors:** Mark J. Siedner, Guy Harling, Anne Derache, Theresa Smit, Thandeka Khoza, Resign Gunda, Thobeka Mngomezulu, Dickman Gareta, Nomathamsanqa Majozi, Eugene Ehlers, Jaco Dreyer, Siyabonga Nxumalo, Njabulo Dayi, Gregory Ording-Jesperson, Nothando Ngwenya, Emily Wong, Collins Iwuji, Maryam Shahmanesh, Janet Seeley, Tulio De Oliveira, Thumbi Ndung'u, Willem Hanekom, Kobus Herbst

**Affiliations:** 1 Africa Health Research Institute, Durban, KwaZulu-Natal, South Africa; 2 Massachusetts General Hospital, Boston, MA, USA; 3 Harvard Medical School, Boston, MA, USA; 4 Institute for Global Health, University College London, London, UK; 5 MRC/Wits Rural Public Health & Health Transitions Research Unit (Agincourt), University of the Witwatersrand, Johannesburg, South Africa; 6 Department of Epidemiology and Harvard Center for Population and Development Studies, Harvard T.H. Chan School of Public Health, Boston, MA, USA; 7 Department of Global Health and Infection, Brighton and Sussex Medical School, University of Sussex, Brighton, UK; 8 Global Health and Development Department, London School of Hygiene & Tropical Medicine, London, UK; 9 KwaZulu-Natal Research Innovation and Sequencing Platform (KRISP)), School of Laboratory Medicine and Medical Sciences, University of KwaZulu Natal, Durban, KwaZulu-Natal, South Africa; 10 Department of Global Health, University of Washington, Seattle, USA; 11 Division of Infection and Immunity, University College London, London, UK; 12 HIV Pathogenesis Programme, The Doris Duke Medical Research Institute, University of KwaZulu Natal, Durban, KwaZulu-Natal, South Africa; 13 Max Planck Institute for Infection Biology, Berlin, Germany; 14 SAPRIN, South African Medical Research Council, Cape Town, South Africa

**Keywords:** Covid-19, Surveillance, South Africa, Screening, Health and Demographic Surveillance System

## Abstract

A coordinated system of disease surveillance will be critical to effectively control the coronavirus disease 2019 (Covid-19) pandemic. Such systems enable rapid detection and mapping of epidemics and inform allocation of scarce prevention and intervention resources. Although many lower- and middle-income settings lack infrastructure for optimal disease surveillance, health and demographic surveillance systems (HDSS) provide a unique opportunity for epidemic monitoring. This protocol describes a surveillance program at the Africa Health Research Institute’s Population Intervention Platform site in northern KwaZulu-Natal. The program leverages a longstanding HDSS in a rural, resource-limited setting with very high prevalence of HIV and tuberculosis to perform Covid-19 surveillance. Our primary aims include: describing the epidemiology of the Covid-19 epidemic in rural KwaZulu-Natal; determining the impact of the Covid-19 outbreak and non-pharmaceutical control interventions (NPI) on behaviour and wellbeing; determining the impact of HIV and tuberculosis on Covid-19 susceptibility; and using collected data to support the local public-sector health response.

The program involves telephone-based interviews with over 20,000 households every four months, plus a sub-study calling 750 households every two weeks. Each call asks a household representative how the epidemic and NPI are affecting the household and conducts a Covid-19 risk screen for all resident members. Any individuals screening positive are invited to a clinical screen, potential test and referral to necessary care – conducted in-person near their home following careful risk minimization procedures. In this protocol we report the details of our cohort design, questionnaires, data and reporting structures, and standard operating procedures in hopes that our project can inform similar efforts elsewhere.

## Introduction

The global emergence of the novel coronavirus, SARS-CoV-2, and the associated pandemic of coronavirus disease 2019 (Covid-19) in humans, has led to substantial morbidity and mortality ^1^. Models project continued risk until effective treatment or vaccines become available, or sufficient immunity is induced in the population to limit transmission ^2, 3^. The transmission and clinical patterns of the pandemic have been well documented in areas of the world where systematic data capture is in place ^4– 7^. However, data from low- and middle-income countries (LMIC) remain scant.

Multiple factors place resource-limited settings at considerable risk of epidemics of respiratory disease. Housing structures, large informal work sectors, high population density (particularly in urban slums and settlements), lack of running water and access to hygiene resources, and a health infrastructure unprepared for diseases requiring advanced medical equipment, all place LMICs at risk of worse outcomes than higher-income settings ^8– 12^. There have long been calls for strengthening public health preparedness in such countries ^13, 14^, but despite some progress ^15^, significant capacity shortfalls remain in many LMICs ^16– 18^. As a result, low case numbers in resource-limited settings should not necessarily be interpreted to reflect a lack of disease transmission. A coordinated system of disease surveillance will be critical to effective prevention, control, and response to the Covid-19 epidemic in LMICs to enable rapid detection, transmission mapping, and non-pharmaceutical interventions, and to inform allocation of scarce prevention and intervention resources.

A unique opportunity for epidemic monitoring within the sub-Saharan Africa region is the presence of longstanding health and demographic surveillance systems (HDSS) ^19– 21^. These routine data collection systems can serve as a vital tool in the public health response to Covid-19. HDSS data are typically collected through interviews that generate population-representative longitudinal cohorts, including information on demographics (births, deaths, migration), health status and behaviours. Historically, they have been conducted in person, however, they have increasingly incorporated telephonic or online data collection ^22, 23^. Additionally, HDSS are well-positioned to evaluate the long-term impacts of the epidemic, by measuring: (i) disease penetration through serologic testing; (ii) causes of death during and after the epidemic through the use of verbal autopsies ^24– 26^; and epidemic impact on economic indicators, and non-Covid healthcare access and health outcomes through ongoing interviews.

This protocol paper describes the program developed by the Africa Health Research Institute (AHRI) to leverage a longstanding HDSS to perform Covid-19 surveillance in a rural, resource-limited setting with very high prevalence of HIV and tuberculosis. In this protocol we illustrate how we have leveraged existing expertise and population-based surveillance infrastructure to conduct comprehensive Covid-19 surveillance in a rural area without existing surveillance capacity for rapidly evolving epidemic disease. We also highlight measures taken to minimize Covid-19 transmission risk while conducting this research. We provide our methods and surveillance tools as potential sources of guidance for others working in similar settings.

## Protocol

### Study aim and objectives

The aims of this study are to conduct active Covid-19 surveillance and to estimate the health and non-health impacts of the Covid-19 epidemic in rural South Africa. To achieve these aims we will implement longitudinal surveillance based on regular telephonic contact with households in a geographically defined population. This surveillance will allow us to meet the following programmatic and scientific objectives:

a. Support the local public health response by the Department of Health (DoH) through screening, testing, case notification and linking Covid-19 cases to care;
b. Describe the incidence and evolution of Covid-19 epidemic in a well-defined population in rural KwaZulu-Natal, including its distribution by age, gender and socio-demographic characteristics;
c. Determine the impact of the Covid-19 outbreak and non-pharmaceutical control interventions on individual behaviour and wellbeing, household socio-economic status and health service delivery;
d. Determine the impact of pre-existing health conditions, including HIV and tuberculosis on Covid-19 susceptibility, clinical presentation and outcomes.

### Study setting

The study will be conducted within the AHRI Population Intervention Platform surveillance area in the uMkhanyakude district of KwaZulu-Natal province. The study area covers approximately 850 km ^2^; it is largely rural with one town of approximately 30,000 residents, and among the lowest-ranked areas in South Africa in terms of socioeconomic status ^27^. A large proportion of the population live with HIV (~20% of men and ~40% of women aged 15–54 in 2017 ^28^). Tuberculosis incidence in the district in 2015 was above the national average of 577 per 100,000 individuals ^29^. Mobile phone ownership in the population is high, 90% of households report owning a phone and in 84% of households there is a valid phone number on record for at least one household member.

AHRI is a member of the South African Population Research Infrastructure Network (SAPRIN), an initiative funded by the National Department of Science and Innovation (DSI) and hosted by the South African Medical Research Council ^30^. SAPRIN standardised the health and demographic surveillance protocols of the three established HDSS sites, in rural KwaZulu-Natal (AHRI), Limpopo and Mpumalanga ^27, 30– 32^. Through SAPRIN, the three HDSS sites agreed on a common Covid-19 surveillance protocol, which each node then adapted to its specific local health priorities and linkage to care pathways.

### Underlying HDSS

Since 2000 AHRI has conducted surveillance of approximately 20,000 households (totalling over 100,000 resident individuals) to capture births, deaths, migrations and household socio-demographics. All households under surveillance are contacted once during each 15-week (triannual) surveillance cycle. All visits were initially face-to-face, but since 2017, two of the three data collection encounters each year have been collected using a telephonic interviewing platform, which allows for high-throughput surveillance. These interviews are conducted with one household senior representative, acting as proxy for all other members. In addition, residents aged 15 years and older are invited to participate individually in an annual biobehavioural survey. AHRI has also embedded data collection clerks within the eleven public health clinics and one district hospital serving the study population. These clerks electronically capture clinic visits and hospital admissions, and link these with the surveillance platform data for all consenting attendees. The population database is also routinely linked to the electronic tuberculosis and HIV treatment registers at these facilities. AHRI holds memoranda of understanding with the Provincial and District DoH that permit extraction of health record data from primary care and hospital sites for linkage to the household surveillance dataset.

### Study design

**Figure 1.**
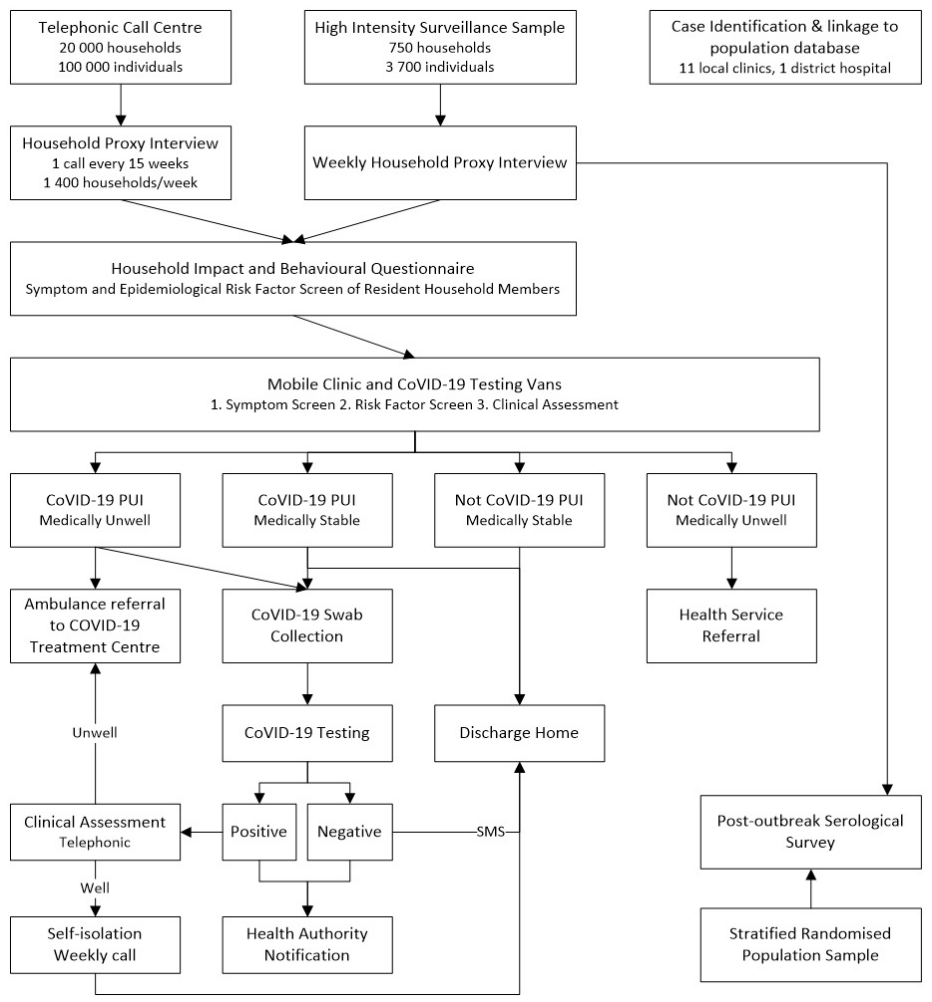
Study flow diagram.

The overall flow of this study is described in Figure 1.

### Household questionnaires

This protocol builds upon the underlying routine health and demographic surveillance. For the duration of the Covid-19 lockdown period (and until it is deemed safe by both the South African government and study investigators), physical household visits have been suspended and replaced by telephonic interviews with the household proxy respondent. In addition to the standard demographic surveillance questions, proxy respondents are asked to answer a Covid-19 questionnaire covering:

a. A screen for symptoms of Covid-19 for each resident household member, completed either by the proxy informant or the household member, if available;
b. Epidemiologic risk factors for Covid-19 including known contacts, healthcare contact, and travel;
c. Household awareness of Covid-19;
d. Behaviour changes in response to the epidemic;
e. Recent visitors to the household and recent travel patterns;
f. Economic and healthcare impacts of Covid-19 non-pharmaceutical interventions on the household;
g. Brief mental health assessments of the household respondent, using the Patient Health Questionnaire-2 and Generalized Anxiety Disorder-2 screening tools ^33, 34^;

The Covid-19 questionnaire takes about 15-20 minutes to administer telephonically, whereas when it is integrated into the household demographic surveillance questionnaire, the complete questionnaire takes about 45 minutes in total.

To improve the temporal resolution of Covid-19 surveillance, a cohort of 750 households (approximately 3,750 individuals) will also be invited to participate in more intensive surveillance, with calls every two weeks, during which all Covid-19 questions are repeated. The intensive household cohort were randomly selected from all households for which at least one member also participated in a recent population-wide HIV, tuberculosis, and non-communicable disease healthcare screening study.

### Individual questionnaires

To ensure that detailed information on reaction to the Covid-19 pandemic is available on the full age-range of residents, we will sample 400 past participants in the healthcare screening study, stratifying by age and gender, and oversampling those with multiple non-communicable health conditions. These individuals will be invited to participate in two-weekly calls to report directly on how the epidemic has affected them, including detailed descriptions of their travel and direct personal contact with others.

Additionally, planned qualitative work will gather in-depth information from a further sub-sample of the study population using telephone interviews to adhere with national social distancing regulations. This will allow an in-depth understanding of perceptions of risks associated with Covid-19, and vulnerabilities experienced by the community. Data collection tools for this have been informed by risk perceptions contributory domains including knowledge, personal risk, severity and probability of possible outcomes, response efficacy as well as precautionary behaviour and use of information sources. Aspects of vulnerability within the tool focus on how likely the respondent thought he or she was to get the disease and aspects they thought made someone vulnerable to COVID-19. Working with local traditional leaders will help identify contextually appropriate solutions to deleterious effects of both Covid-19 epidemic and non-pharmaceutical interventions used to mitigate its effects.

### Clinical screening of persons under investigation

If a household member screens positive as a potential person under investigation (PUI) for Covid-19, based on the current South African Covid-19 screening guidelines (see full screening algorithm in Supplementary Material ^35^), the individual is referred to an AHRI Covid-19 mobile testing centre for screening and a possible diagnostic test. These mobile testing centres will come to within walking distance of participant homes. The testing centres are staffed by AHRI research assistants and senior professional nurses and include strict infection control and personal protective equipment procedures ^35^. A research assistant performs a symptom and epidemiologic screen to confirm PUI status. Those meeting PUI criteria are referred to a professional nurse who conducts a clinical examination for triage, including temperature, respiratory rate, and oxygen saturation monitoring. Nurses then complete a testing and referral questionnaire (see Supplementary Material ^35^) on a tablet computer using web-based REDCap forms ^36^. Responses trigger assignment to one of the following statuses:

a. PUIs who meet the case definition, and require clinical care will undergo Covid-19 testing and specimen collection, and then be referred to a local Covid-19 treatment facility;
b. PUIs who meet the case definition, but who do not require clinical care, will undergo Covid-19 testing and specimen collection, and then be referred home for self-isolation after being given self-isolation and general Covid-19 educational materials;
c. Individuals who do not meet the case definition for testing and require clinical care will be referred to a local health care facility;
d. Individuals who do not meet the case definition and do not require clinical care will be sent home with Covid-19 information material.

### Specimen collection and testing

Specimen collection consists of nasopharyngeal swab and oropharyngeal swabs placed together into a cryovial containing 2 ml viral transport medium labelled with a unique DataMatrix ^37^ barcode and electronically logged. For young children a single nasal mid-turbinate swab (collected from both nostrils) will replace the nasopharyngeal and oropharyngeal swabs. Specimens will be stored and shipped at 2–8°C to the AHRI Diagnostic Laboratory in Durban within 24 hours of sample collection, for processing and testing.

On receipt in the AHRI Diagnostic Laboratory, the specimens will be registered and tracked on the Laboratory Information Management system (LIMS) ^38^ and undergo inactivation (at 60°C for 30 minutes). Following inactivation, extraction will be performed either manually using the QIAamp® Viral RNA kit (Qiagen, Germany) or on the Chemagic ^TM^ 360 Nucleic Acid Extractor (Perkin Elmer, Waltham, MA, USA) using Chemagic ^TM^ Viral DNA/RNA 300 Kit H96 (Perkin Elmer, Waltham, MA, USA). SARS-CoV-2 templates will be generated using the TaqMan ^TM^ 2019-nCoV Assay Kit v1 or v2 (Applied Biosystems, Foster City, CA, USA) and run on The Applied Biosystems QuantStudio 5 or 7 Real-Time PCR System by the KwaZulu-Natal Research Innovation and Sequencing Platform (KRISP) or by AHRI on a CFX ^TM^ Touch Real-Time System (Bio-Rad (Hercules, CA, USA) or Abbott m2000rt).

We will use comparative Ct to analyse 2019-nCoV assay data, with Ct <37 regarded as positive (CoV-2 RNA detected), Ct ≥37 but <40 considered indeterminate, and Ct ≥40 as negative. Indeterminate results will be repeated from the amplification process and if found to be indeterminate, the sample re-extracted and amplified; if the sample remains indeterminate it will be reported as suspected positive/positive. Positive cases with a Ct <20 will be genotyped using the ARTIC network SARS-CoV-2 whole genome sequencing (WGS) protocol at KRISP ^39^. WGS will be assembled using Genome Detective ^40^ and analysed using phylogenetic software applications, such as BEAST ^41^ and Nextstrain ^42^. All genomes will be analysed with over 12,000 WGS publicly available in the GISAID database ^43^.

### Results reporting and disease monitoring

The SARS-CoV-2 test results will be electronically transferred to the study database and presented for clinical review to the study physician.

a. PUIs testing negative for SARS-CoV-2 will be informed of their results via short message service (SMS);
b. PUIs testing positive for SARS-CoV-2 will be called by a study physician to be informed of their results. Covid-19 cases will be followed longitudinally with phone calls every three days for clinical monitoring until resolution of symptoms. Those with worsening symptoms will be referred to the AHRI mobile testing centre for further attention and, if warranted, referral to the regional Covid-19 treatment centre.

All Covid-19 test results will be submitted to the national notifiable medical conditions web portal; a system-generated PUI (including a contacts list) form will also be submitted to the local public health service.

After the Covid-19 outbreak and upon resumption of the parent AHRI surveillance protocol, which provides for an annual dried blood spot collection from resident individuals age 15 years and above, the protocol provides for measurement of SARS-CoV-2 seroprevalence, which will be conducted to determine contributions of asymptomatic disease to the epidemic.

All participants with positive COVID-19 test results will be considered for enrolment in a related study titled, "Consequences of HIV and TB Co-Infection on COVID-19 Disease Dynamics, Severity and Immune Responses," which is a longitudinal disease characterization and sample collection protocol to establish mechanistic interactions between HIV, TB and COVID-19.

### Data analysis and sample size

We will collect and report Covid-19 surveillance data in real-time to local partners, including the DoH and the AHRI Community Advisory Board. At the conclusion of each week, we will report the following indicators to key stakeholders:

1. Number of households screened by phone call;
2. Number of individuals screened (through proxy);
3. Proportion of households contacted and consented;
4. Proportion of households and individuals with a positive case definition screen;
5. Number of PUI screened at Covid-19 clinics;
6. Number of Covid-19 tests conducted;
7. Number and proportion of Covid-19 tests resulting positive;
8. Number of individuals referred to clinic for moderate/severe Covid-19 disease;
9. Population-representative prevalence of Covid-19 overall and by age and sex.

At the conclusion of each 15-week surveillance block, we will additionally conduct a total population Covid-19 epidemiologic summary, including age, sex, and HIV-serostatus stratified Covid-19 prevalence and prevalence of moderate/severe disease. This project is designed to conduct surveillance of the entire HDSS population area and thus is not powered to test a specific hypothesis. However, with a total population sample size of 100,000, and assuming 60% participation in each 15-week block from at least one household member, we will have substantial power to estimate overall and stratified Covid-19 prevalence estimates within age and sex strata ( Table 1).

**Table 1.** The 95% confidence intervals for prevalence estimates for surveillance platform based on sub-group and total population prevalence of 1 and 5%.

Sex	Age Band	Population Size	95% confidence interval for 1% prevalence *	95% confidence interval for 5% prevalence *
Female	<19 years	21,922	0.87 – 1.11%	4.71 – 5.29%
Male	<19 years	22,041	0.87 – 1.13%	4.71 – 5.30%
Female	20–49 years	23,853	0.87 – 1.13%	4.73 – 5.29%
Male	20–49 years	17,220	0.86 – 1.16%	4.67 – 5.33%
Female	>49 years	11,241	0.82 – 1.20%	4.60 – 5.42%
Male	>49 years	4,958	0.75 – 1.33%	4.41 – 5.64%
Total Pop	--	101,235	0.94 – 1.06%	4.87 – 5.13%

*Confidence intervals estimated with the exact method to account for small proportion sizes.

The data collected will provide the opportunity to conduct a wide range of analyses. In particular, the 750-household cohort will allow local epidemic dynamics to be carefully mapped to changes in behaviour, individual wellbeing and household economic and healthcare access, using methods including time-to-event and interrupted time series analysis. Existing data, both collected by AHRI and routinely collected by others and linked to AHRI unique identifiers, will allow comparison of residents’ current situation with that in previous years.

***Data management***. Data collected in this study will be centrally stored on AHRI’s secure data servers. Data will be collected through interviews at the call centre on central server using Survey Solutions ^44^, and in the field on encrypted tablet computers using REDCap, and then securely uploaded to a central server. Collected specimens will be logged on CRF system and control lists generated to verify that all specimens have arrived at the laboratory. These lists will be electronically transferred to LIMS. An electronic study dashboard will assist operational teams in monitoring study progress. Database rules and constraints will be enforced on the master database to ensure that only valid data is stored. Personally identifiable data will be restricted to separate database tables accessible only to data management staff – these will never be shared with the research team.

### Risks and benefits for participants

The protocol adheres to the provisions of the Covid-19-specific National Infection Prevention and Control Strategic Framework ^45^, as well as research conduct guidance by national and international research oversight bodies. The main risk to study activities and participation is of SARS-CoV-2 transmission from in-person interactions between staff and study participants (i.e., at testing centres). Following a risk assessment ^35^, we have implemented multiple control measures to minimize this risk, including: physical barriers; physical distancing; regular hand hygiene; and careful training on and use of standard operating procedures. Should it become impossible to comply with these provisions due to personal protective equipment supply shortages, case load, or other unforeseen challenges, surveillance will be paused, scaled back or terminated.

### Consent and assent

This protocol builds on the consent procedures in place for the household surveillance protocol it is embedded in. All households contacted for this study have previously provided written consent to: (i) be approached via a telephone call to be asked to participate in research studies; (ii) electronic linkage of surveillance study records with their medical records; and (iii) the sharing of anonymized data in data repositories accessible to the broader scientific community.

In this study, all participants are asked to provide verbal informed consent ^35^ prior to telephone interviews, which will be recorded in accordance with Covid-19 specific guidance from the University of KwaZulu-Natal Biomedical Research Ethics Committee ^46^. Additionally, verbal consent for specimen collection and testing will be obtained (and recorded on the electronic case form) at the specimen collection point. Verbal recording of consent will remove the danger of transmitting SARS-COV-2 via the data collection tablet or a stylus. Any potential participants aged under 18 and not emancipated will be asked to provide recorded verbal assent after a responsible adult has provided informed consent.

### Community engagement

The protocol was presented to both the DoH and the AHRI Community Advisory Board management committee and approved by both prior to submission for ethical approval. Bulk SMS messages are used to communicate Covid-19 information and study specific arrangements to the research population. Local radio stations will broadcast information on the study and feature interviews with study staff.

### Ethical approval

The protocol received ethical approval from the University of KwaZulu-Natal Biomedical Research Ethics Committee on 2 April 2020 under reference BE290/16 and from the KwaZulu DoH Research Committee.

### Dissemination policy

The key findings of this study will be prepared for dissemination as rapidly as possible, given the urgency of the Covid-19 epidemic. These will be provided to the DSI and other government decision-makers at national and provincial levels, including the provincial DoH. They will also be submitted to open-access pre-print servers and as open-access articles to peer-reviewed journals.

### Study limitations

One limitation is the possibility of not re-interviewing the participant in the next round. On average, 12.0% (range 5.7%–16.9%) of HDSS participants are not re-interviewed the next year due to out migration, death, loss to follow-up or refusal. Over 80% of these losses are due to outmigration; however, 80% of these outmigrants remain household members, and are therefore accessible to collect outcome data. Additionally, for an acute infection with a relatively short symptom duration, the 15 weeks between surveillance contacts means that some proportion of cases will be missed. This limitation is mitigated to some extent by the two-weekly surveillance of 750 households, but the analytical power of this cohort will depend on the cumulative incidence of Covid-19 in the overall study population. Moreover, our study depends on symptomatic screening and proxy responses by heads of households, which will also be susceptible to under-reporting ^47– 49^. Proxy-based case identification will be augmented by documentation of cases from the study community presenting at the eleven primary health care clinics and one district hospital in the population study catchment area. Our ability to conduct a post-outbreak seroprevalence study will allow for detection of missed cases in this surveillance program, and ultimately serve a complementary role in epidemic description in this population.

## Discussion

Widespread SARS-CoV-2 testing for surveillance and case identification has been established as a major priority in the epidemic response. Although testing capacity has been limited in many areas of the world, HDSS provide a pre-existing structure in resource-limited settings that is purpose-built for surveillance, with both expertise and infrastructure for conducting population-based surveys.

We have leveraged a HDSS to develop and implement a Covid-19 surveillance program in rural South Africa. In doing so, we focused on the following major considerations to ensure both optimal scientific methods and the safety of our staff and study population:

1. Total-population sampling, with enhanced surveillance of random sub-samples for early identification of high transmission areas and groups;
2. Phone-based screening surveillance to minimize in-person contact;
3. Limiting all in-person interactions to persons under investigation;
4. Strict adherence to personal protective equipment use, well-trained staff using standardized operating procedures and risk mitigation plans, and hygiene measures in line with national and international guidelines;
5. Laboratory procedure conducted in a clinical research laboratory within good laboratory clinical practice guidelines;
6. Partnership with DoH and other government departments for case detection, reporting, contact tracing and to ensure all procedures fall within governmental guidance;
7. Real-time reporting of surveillance results to inform epidemic response and resource allocation.
8. Leveraging routinely collected data such as household socio-economic status, individual employment and education status and linkage to individual health service data to determine the impact of the Covid-19 pandemic on household socio-economic status and health care delivery.

Herein we report the details of our cohort design, questionnaires, data and reporting structures, and standard operating procedures in hopes that our project can be used to help inform other similar programs elsewhere in the region.

### Future data availability

A core set of the Covid-19 surveillance indicators and data will made available for the three SAPRIN nodes in the publicly accessible SAPRIN data repository ( http://saprindata.samrc.ac.za/index.php/catalog). AHRI-specific data will be disseminated through the AHRI data repository ( https://data.africacentre.ac.za/) within six months from the conclusion of the first 15-week surveillance cycle. Both datasets will be linkable to existing core demographic surveillance data.

## Data availability

### Underlying data

No underlying data are associated with this article.

### Extended data

Zenodo: Protocol: Leveraging a demographic and health surveillance system for Covid-19 Surveillance in rural KwaZulu-Natal Supplementary Material. https://doi.org/10.5281/zenodo.3992527 ^35^

This project contains the following extended data:

- Suppl Mat 1.docx (South African national Covid-19 screening algorithm)
- Suppl Mat 2.docx (Standard Operating Procedures)
- Suppl Mat 3.docx (Questionnaires)
- Suppl Mat 4.docx (Risk Assessment)
- Suppl Mat 5.docx (Informed consent wording)

Data are available under the terms of the Creative Commons Attribution 4.0 International license (CC-BY 4.0).
