# Supplementary material for: Protocol: Leveraging a demographic and health surveillance system for Covid-19 Surveillance in rural KwaZulu-Natal
Source: Wellcome Open Res. 2020 Aug 25;5:109. Originally published 2020 May 29. [Version 2] doi: 10.12688/wellcomeopenres.15949.2 (PMC7424917; doi:10.12688/wellcomeopenres.15949.2)
Supplement: Peer review history

# Peer review: Reviewer response for version 1

**DOI:** 10.21956/wellcomeopenres.17493.r39709

**Published:** 2020-08-12

**Authors:** Jacques Emina

**Affiliations:** 1 Department of Population and Development Studies, University of Kinshasa, Kinshasa, Congo; 2 Population and Health Research Institute (PHERI), Kinshasa, Congo

Leveraging a demographic and health surveillance system for Covid-19 Surveillance in rural KwaZulu-Natal is an important implementation research programme in low-and-middle countries. This programme will allow monitoring and documenting COVID-19 in the context of data paucity. The project will also allow to evaluate the effect of public policies and other interventions in fighting the pandemic. The project is well designed and is build on an existing data platform and well known context. However, the protocol does not provide information on the phone coverage in the Demographic Surveillance Area. Do all households have a land line and/or mobile phone? What are the length of different questionnaires listed? What could be the influence of number of questionnaires (and questions) on the response rate, quality of answer and risk of abandon?

 I recommend indexing of this manuscript with minor comments described above.

Is the study design appropriate for the research question?

Yes

Is the rationale for, and objectives of, the study clearly described?

Yes

Are sufficient details of the methods provided to allow replication by others?

Yes

Are the datasets clearly presented in a useable and accessible format?

Yes

Reviewer Expertise:

Population and Health, evaluation, data sciences and Health Systems

I confirm that I have read this submission and believe that I have an appropriate level of expertise to confirm that it is of an acceptable scientific standard.

# Response

**Published:** 2020-08-20

**Authors:** Kobus Herbst

**Affiliations:** DSI-MRC South Africa Population Research Infrastructure Network, South Africa

We thank the reviewer for the positive review. We have submitted a new version of the protocol description with details on the phone coverage in the surveillance area and also provided information of the average time it takes to complete the various questionnaires used.

# Peer review: Reviewer response for version 1

**DOI:** 10.21956/wellcomeopenres.17493.r39141

**Published:** 2020-07-09

**Authors:** Izukanji Sikazwe

**Affiliations:** 1 Centre for Infectious Disease Research in Zambia (CIDRZ), Lusaka, Zambia

Overall an appropriate protocol whose study will provide meaningful insights on the extent of and the impact of Covid-19 on behaviour and wellbeing and susceptibility to Covid-19 due to HIV/TB in a rural population that could be generalised to similar settings in the region.

 The investigators leverage an existing health and demographic surveillance system built over several years in a rural setting in KZN with a high prevalence of HIV and TB to perform Covid-19 surveillance. The surveillance system is well developed and multiple surveys have been conducted within this community making the proposed work feasible.

 The protocol objectives are clear and how these objectives will be achieved is well described. The study population and how it will be accessed is documented and includes details on how risk to Covid-19 for study staff or the population will be mitigated. They further show how this work will supplement DOH activities and will ensure that any potential Covid-19 participants are linked to care.

 The protocol however needs additional information on how they will determine the impact of Covid-19 outbreak and non-pharmaceutical control interventions on individual behaviour and wellbeing, household socio-economic status and health service delivery. Additional information on the planned qualitative work, methodology, tools to be used would be helpful to enable others replicate this work.

Is the study design appropriate for the research question?

Yes

Is the rationale for, and objectives of, the study clearly described?

Yes

Are sufficient details of the methods provided to allow replication by others?

Partly

Are the datasets clearly presented in a useable and accessible format?

Not applicable

Reviewer Expertise:

HIV patient outcomes implementation science

I confirm that I have read this submission and believe that I have an appropriate level of expertise to confirm that it is of an acceptable scientific standard.

# Response

**Published:** 2020-08-20

**Authors:** Kobus Herbst

**Affiliations:** DSI-MRC South Africa Population Research Infrastructure Network, South Africa

We thank the reviewer for the positive review. We have submitted a new version of the protocol description that clarifies the points raised by the reviewer regarding how we plan to determine impact and we also provide more details on the qualitative research component.
